# Comparison of Trifecta and Pentafecta Outcomes between T1a and T1b Renal Masses following Robot-Assisted Partial Nephrectomy (RAPN) with Minimum One Year Follow Up: Can RAPN for T1b Renal Masses Be Feasible?

**DOI:** 10.1371/journal.pone.0151738

**Published:** 2016-03-17

**Authors:** Dae Keun Kim, Lawrence H. C. Kim, Ali Abdel Raheem, Tae Young Shin, Ibrahim Alabdulaali, Young Eun Yoon, Woong Kyu Han, Koon Ho Rha

**Affiliations:** 1 Department of Urology, CHA Seoul Station Medical Center, CHA University, CHA Medical School, Seoul, Republic of Korea; 2 Department of Urology, School of Medicine, Graduate School, Hanyang University, Seoul, Republic of Korea; 3 Department of Urology, Royal Prince Alfred Hospital, Missenden road, Camperdown, Sydney, NSW, Australia; 4 Department of Urology, Tanta University, Tanta, Egypt; 5 Department of Urology, Chuncheon Sacred Hospital, College of Medicine, Hallym University, Chuncheon, Republic of Korea; 6 Department of Urology, Urological Science Institute, Yonsei University College of Medicine, Seoul, Republic of Korea; Baylor College of Medicine, UNITED STATES

## Abstract

**Purpose/Objectives:**

To investigate the feasibility of RAPN on T1b renal mass by assessment of Trifecta and Pentafecta rate between T1a and T1b renal mass.

**Materials/Methods:**

We retrospectively reviewed the medical records of 277 cases of RPN performed from 2006 to 2015. Sixty patients with clinically T1b renal masses (> 4cm and ≤ 7 cm) were identified, and from 180 patients with clinically T1a renal mass, 60 patients were matched with T1b renal mass by propensity score. Tumor complexity was investigated according to R.E.N.A.L nephrometry score. “Pentafecta” was defined as achievement of Trifecta (negative surgical margin, no postoperative complications and warm ischemia time of ≤ 25 minutes) with addition of over 90% estimated GFR preservation and no chronic kidney disease stage upgrading at 1 year postoperative period. Propensity score matching was performed by OneToManyMTCH. Logistic regression models were used to identify the variables which predict the Trifecta, and Pentafecta ac.

**Results:**

Preoperative variables (age, sex, body mass index, ASA score) were similar between T1a and T1b after propensity score matching. The median R.E.N.A.L. nephrometry score was 8 vs 9 for T1a and T1b respectively (p<0.001). The median warm ischemia time was 20.1 min vs 26.2 min (p<0.001). Positive surgical margin rate was 5% vs 6.6% (p = 0.729) and overall complication rate of 13.3%. vs 15% (p = 0.793). The rate of achievement of Trifecta rate were 65.3% vs 43.3% (p = 0.017) and Pentafecta rate were 38.3% vs 26.7% (p = 0.172). For achievement of Pentafecta, R.E.N.A.L nephrometry score (HR 0.80; 95% CI (0.67–0.97); p = 0.031) was significant predictor of achieving Pentafecta. Subanalyis to assess the component of R.E.N.A.L nephrometry score, L component (location relative to the polar lines, HR 0.63; 95% CI (0.38–1.03); *P* = 0.064) was relatively important component for Pentafecta achievement.

**Conclusions:**

The rate of Pentafecta after RAPN was comparable between T1a and T1b renal masses. RAPN is a feasible modality with excellent long term outcome for patients with larger renal mass (cT1b).

## Introduction

Robot-assisted partial nephrectomy (RAPN) has been increasingly utilized in the treatment of localized renal tumors. Recently, European Association of Urology (EAU) guidelines recommend partial nephrectomy (PN) as the standard procedure for larger renal masses >4 cm (T1b), in technically feasible cases [1]. The adoption of robotic platform for PN has been rapidly increased for the treatment of larger, and complicated renal masses [2]. Recent meta-analysis regarding perioperative outcomes of RAPN and laparoscopic partial nephrectomy (LPN) have supported the evidence for the adoption of RAPN [3, 4]. Based on these results, we suggested to provide the equivalent oncological, functional outcomes as well as perioperative outcomes for T1b renal mass.

The ‘Trifecta’, the term for describing the achievement of no complication, negative surgical margin, and warm ischemia time (WIT)< 25 min is the key surrogate for success of the PN [5]. Recently, with additional information of long term renal function, the new concept of ‘Pentafecta’ was introduced to prove the success of renal function preservation after PN [6]. However, there is no data available regarding Pentafecta or Trifecta of RAPN series including T1b renal tumors. Herein, we investigate the comparative outcomes of Trifecta and Pentafecta in cases of RAPN (T1a vs T1b renal tumors) to offer a wider range of indications and potential expanding applicability of RAPN for more larger tumors.

## Materials and Methods

### Study patients

After obtaining institutional review board approval by human research protection center, severance hospital yonsei university health system (2014-0091-001), the patient records was anonymized and de-identified prior to analysis. We retrospectively analyzed the data of 277 patients who underwent RAPN between Sep. 2006 and Mar. 2015 by single surgeon (KHR), in a high volume institute.

From this cohort, RAPN for renal mass of ≤7 cm were included in this study. The patients with solitary kidney (3 cases), bilateral renal mass (4 cases), cT≥3 (9 cases), or incomplete data (21 cases) were excluded. We investigated clinical data of 180 patients with cT1a renal mass, and 60 patients with cT1b renal mass. Of 180 patients with clinically T1a renal mass, 60 patients were matched with the patients with T1b renal mass using propensity score.

The propensity score of each patient was calculated using preoperative variables which included age, gender, ASA score, laterality of mass, body mass index (BMI). The discrimination and calibration of the propensity score model were assessed by C statistics and the Hosmer–Lemeshow test. The model had a C statistic of 0.771 and a Hosmer–Lemeshow goodness-of-fit *P* value of 0.907.

RAPN was performed via transperitoneal approach using the da Vinci Surgical System (Intuitive Surgical, Sunnyvale, CA, USA). Surgical technique of RAPN is same as previously reported [7].

### Clinical assessment

Patients clinical characteristics [age, gender, BMI, preoperative renal function, comorbidity, chronic kidney disease stage), tumor characteristics [R.E.N.A.L nephrometry score], intraoperative data [operative time, warm ischemia time (WIT), estimated blood loss (EBL)], and pathology results [surgical margin status, histology type], and postoperative renal function were investigated.

The Pentafecta was defined as achievement of Trifecta (negative surgical margin, no postoperative complications (Clavien-Dindo≥2) and warm ischemia time of ≤ 25 minutes) with addition of over 90% estimated GFR preservation (eGFR) and no chronic kidney disease (CKD) stage upgrading at 12 months postoperatively [6]. CKD upstaging was defined as upstaging of CKD stage to III,IV,V [8]. Upstaging form CKD stage I to II was regarded as clinically non significant. Postoperative complication was graded according to the modified Clavien-Dindo classification [9]. Renal function was analyzed preoperatively and postoperatively to 12 months. The eGFR rate was estimated by modification of diet in renal disease (MDRD) equation formula [10]. The stage of CKD was based on the National Kidney Foundation Kidney Disease Outcome Quality Initiative Classification [11]. A positive surgical margin was defined as tumor cell presents on the inked parenchymal margin.

Primary end point was Pentafecta and Trifecta accomplishment rate of T1a and T1b renal mass.

### Statistical analysis

Continuous variables were presented as median and interquartile range (IQR) values. Propensity score matching was performed by OneToManyMTCH. Logistic regression models were used to identify the variables which predict the Trifecta, and Pentafecta accomplishment. Variable with P-value <0.10 on univariable analysis was investigated for multivariable model. A two-sided p value <0.05 was deemed statistically significant. Analysis was performed using the SAS^®^ version 9.2(SAS Institute, Cary, NC, USA).

## Results

### Baseline characteristics

Of 240 patients with clinical T1 renal mass, 60 patients with T1a renal mass were matched to those with T1b renal mass using propensity score matching. Table 1 provides demographic characteristics of the total cohort treated by RAPN before and after matching process. No significant differences were indentified between two groups (T1a vs T1b) for age, sex, BMI, laterality of mass, and ASA score.

**Table 1:** Clinical characteristics of patients with T1a, T1b renal mass.

Variables	Entire cohort			Propensity-score matched cohort		
	T1a (N = 180)	T1b (N = 60)	*P*-value	T1a (N = 60)	T1b (N = 60)	*P*-value
Male	134	33	0.03	36	43	0.178
Age, yrs. (IQR)	51 (45–62)	49.5 (39.8–62)	0.532	52 (46–62)	49.5 (39.8–62)	0.355
BMI, kg/m^2^ (IQR)	23.4 (21.2–25.3)	24.7 (22.7–27.7)	0.103	25 (22.9–27.2)	24.7 (22.7–27.7)	0.531
ASA score category			0.037			0.538
1	130	33		34	33	
2	45	24		24	24	
3	5	3		2	3	
Hypertension	57	15		18	15	
Diabetes mellitus	35	7		13	7	
Median tumor size, cm (IQR)	2.6 (1.8–3.7)	5 (4.5–5.9)	<0.001	2.6 (2.1–3.4)	5 (4.5–5.9)	<0.001
Rt. laterality, n (%)	118 (65.5)	31 (51.6)	0.082	35 (58.3)	31 (51.6)	0.161
Median R.E.N.A.L. score (IQR)	7 (6–9)	9 (8–10)	<0.001	8 (6–9)	9 (8–10)	<0.001
Pathology				Clear cell: 45	Clear cell: 45	
				Papillary cell: 1	Papillary cell: 3	
				Chromophobe: 3	Chromophobe: 2	
				Cystic RCC: 2	Cystic RCC: 3	
				AML: 7	AML: 7	
				Oncocytoma: 2		

Median renal mass size for the T1a and T1b cohorts was 2.6 cm and 5 cm in diameter, respectively (*P*<0.001). The complexity of the renal mass evaluated by R.E.N.A.L. nephrometry score was higher in T1b, 8 vs 9 (*P* <0.001).

### Perioperative outcomes

The perioperative outcomes are shown in Table 2. Median operation time was similar between two groups, 150 min for the T1a and 165.5 min for the T1b group; *P* = 0.261. Median EBL was 300 cc (T1a), 426 cc (T1b); *P* = 0.242. The median WIT was 20.1 min (T1a), 26.2 min (T1b); *P* <0.001. 18.3% of the patients with T1a renal mass and 53.3% with T1b had WIT over 25 min. Overall postoperative complication rates (Clavien Dindo≥2) were comparable between two groups; 13.3% (T1a) vs 15% (T1b); *P* = 0.793 (Table 2). For T1a patients, one patient required transfusion, one patient had pseudoaneurysm required angioembolization, and 6 patients had fever. For T1b patients, three patients required transfusion, one patient had arterio-venous fistula required angioembolization, one patient had perirenal urinoma, and 4 patients had fever.

**Table 2:** Oncological, perioperative, functional outcomes after robot-assisted partial nephrectomy.

Variables	T1a	T1b	*P*-value
Positive surgical margin	3 (5)	4 (6.6)	0.729
WIT>25 min, n (%)	11 (18.3)	32 (53.3)	<0.001
Complications, n (%)	8 (13.3)	9 (15)	0.638
Clavien-Dindo II	7 (11.6)	7 (11.6)	
Clavien-Dindo III	1 (1.6)	2 (3.3)	
Clavien-Dindo IV	0 (0)	0 (0)	
Operation time, min (IQR)	150 (108–180)	165.5 (130–217)	0.261
EBL, mL	300 (162.5–515)	425 (162.5–700)	0.242
Median preoperative eGFR, mL/min/1.73 m^2^ (IQR)	90 (78.1–98.7)	85 (75–99.5)	0.718
Median percentage of eGFR preservation (IQR)	83.9(74.4–96.0)	84.5 (72.9–99.6)	0.492
90% eGFR preservation, n (%)	30 (50)	20 (33)	0.021
Upstaged to CKD III-V postoperatively, n (%)	9 (15)	10 (16.6)	0.874
Trifecta achievement, n (%)	39 (65.3)	26 (43.3)	0.017
Pentafecta achievement, n (%)	23 (38.3)	16 (26.7)	0.172

### Renal function outcome

Preoperative renal function data was not significantly different between two groups; 90 mL/min/1.73 m^2^, 85 mL/min/1.73 m^2^ respectively (*P* = 0.718). Median percentage of eGFR preservation on one year postoperative period was 83.9% (T1a) and 84.5% (T1b), respectively (*P* = 0.492). 50% (T1a) and 33% (T1b) achieved over 90% eGFR preservation (*P* = 0.021). There was no difference in the rate of upstaging to CKD III-V 16.6% (*P* = 0.874). Pathology result of histology distribution revealed in Table 2. Positive surgical margin rate was 5% (T1a) vs 6.6% (T1b) (*P* = 0.729).

### Trifecta and Pentafecta rate

The rate of achievement of Trifecta for T1a and T1b renal mass was 65.3%, 43.3%, respectively (*P* = 0.017), and the rate of achievement of Pentafecta was 38.3%, 26.7%, respectively (*P* = 0.172). On multivariable regression analysis of T1b renal mass for achievement of Trifecta, tumor size (hazard ratio (HR) 0.42; 95% confidence interval (CI) (0.18–0.93); *P* = 0.041), operation time (HR 0.98; 95% CI (0.97–0.99); *P* = 0.011) were significant predictive factors, and for achievement of Pentafecta, R.E.N.A.L nephrometry score (HR 0.80; 95% CI (0.67–0.97); *P* = 0.031) was a significant predictor of achieving Pentafecta (Tables 3 and 4). Subanalyis to assess the component of R.E.N.A.L nephrometry score, L component (location relative to the polar lines, HR 0.63; 95% CI (0.38–1.03); *P* = 0.064) was relatively important component for Pentafecta achievement.

**Table 3:** Logistic regression analysis of factors associated with trifecta achievement after robot-assisted partial nephrectomy on T1b patients

	Univariable (unadjusted HR)			Multivariable (adjusted HR)		
Variable	HR	95% CI	*P*-value	HR	95% CI	*P*-value
Age (years)	0.98	0.94–1.02	0.276			
BMI (kg/m^2^)	1.03	0.88–1.19	0.707			
Tumor size (cm)	0.44	0.19–0.97	0.042	0.42	0.18–0.93	0.041
Operation time (min)	0.98	0.97–0.99	0.009	0.98	0.97–0.99	0.011
EBL (mL)	0.99	0.99–1.00	0.079	0.97	0.98–1.01	0.16
Preoperative GFR (mL/min/1.73m^2^)	1.01	0.98–1.03	0.675			
R.E.N.A.L score	0.88	0.64–1.21	0.419			
E score component	0.99	0.59–1.67	0.973			
N score component	0.73	0.45–1.19	0.215			
L score component	0.74	0.46–1.18	0.200			
Per 100 cases						
2^nd^ vs 1^st.^	1.97	0.56–6.97	0.289			
3^rd^ vs 1^st^	3.36	0.71–15.84	0.126			

HR = hazard ratio; CI = confidence interval

**Table 4:** Logistic regression analysis of factors associated with pentafecta achievement after robot-assisted partial nephrectomy on T1b patients.

	Univariable (unadjusted HR)			Multivariable (adjusted HR)		
Variable	HR	95% CI	*P*-value	HR	95% CI	*P*-value
Age (years)	0.98	0.94–1.02	0.432			
BMI (kg/m^2^)	1.01	0.85–1.19	0.911			
Tumor size (cm)	0.56	0.23–1.34	0.193	0.61	0.24–1.21	0.242
Operation time (min)	0.99	0.97–1.01	0.123	0.97	0.93–1.03	0.212
EBL (mL)	0.99	0.99–1.00	0.245			
Preoperative GFR (mL/min/1.73m^2^)	0.99	0.97–1.02	0.743			
R.E.N.A.L score	0.72	0.51–1.03	0.021	0.80	0.67–0.97	0.031
E score component	0.66	0.34–1.17	0.154			
N score component	0.75	0.46–1.23	0.253			
L score component	0.63	0.38–1.03	0.061			
Per 100 cases						
2^nd^ vs 1^st.^	1.62	0.37–7.16	0.522			
3^rd^ vs 1^st^	3.33	0.61–18.15	0.161			

HR = hazard ratio; CI = confidence interval

## Discussion

The application of robotic platform has been increased in the treatment of renal mass [3]. Recently, the adoption of robotic platform has allowed more complex and challenging cases for PN [12]. The achievement of success on PN could be assessed by Trifecta rate, and recently, the concept of Pentafecta rate was introduced [6]. Improving the rate of Pentafecta should be the main goal on performing RAPN.

In this study, we have investigated the Trifecta, Pentafecta rate of T1a and T1b renal mass, and compared these two groups. We also investigated the predictive factors for achievement of Trifecta and Pentafecta rate. Our study showed that rate of achievement of Trifecta of T1a, T1b renal mass was significantly higher in T1a group (65.3% vs 43.3%), however, Pentafecta rate was statistically comparable between two groups that of 38.3%, 26.7%, respectively. Tumor size and operation time were significant predictive factors of Trifecta achievement. The R.E.N.A.L. nephrometry score was a significant predictive factor of Pentafecta achievement.

There is a huge variation in terms of the rate on achievement of Trifecta, ranging from 32% to 81% [5, 6, 11, 13]. The variation is caused by many factors such as surgical techniques, surgeon’s experience and different definition of Trifecta on PN cases. Trifecta represents a surrogate measure of surgical quality and has been increasingly used as a standardized tool to compare outcomes. There is no standard definition of Trifecta but the core concept remains the same; oncological outcome, functional preservation, and complication free safety. For simplification of reporting the long term outcomes of PN, we used Pentafecta rate to evaluate oncologic outcomes (surgical margin status), complication rate (Clavien-Dindo≥2), immediate functional outcomes (WIT), and long term functional outcomes (90% eGFR preservation, upstage to CKD III-V).

In this study, oncological outcome has been assessed by surgical margin status on final pathology. However, surgical margin status does not reflect the actual long-term oncologic outcomes. In our study overall 5.8% PSM rate was identified including T1b cases, which was comparable with other literature [13]. On safety outcomes, the severity of complication was evaluated according to Clavien-Dindo classification. In the present study, Clavien-Dindo≥2 cases were considered to be clinically significant complication.

Evaluation of renal functional outcome could be affected by several factors. Preoperative variables such as age, sex, preoperative renal function, and intraoperative variables such as WIT, the volume of parenchymal preservation might impact on renal function after PN. WIT is an important surrogate which is shown to influence immediate renal function For the assessment of long term functional outcome, we have included two additional criteria; postoperative eGFR preservation and no CKD upstaging. The threshold of 90% preservation of eGFR is based on published literature regarding functional outcome after PN [14]. We defined significant CKD upstaging to CKD stage III-V. CKD upstaging from I to II has been regarded as clinically insignificant. The combination of CKD upstaging and 90% eGFR preservation could reflect the long term renal functional outcome of PN. Therefore, by combination of Trifecta with two additional long term functional parameters, Pentafecta achievement could facilitate in assessing the precise long term outcome following PN.

Porpiglia et al. [15] assessed 206 patients who underwent LPN using margins, ischaemia and complication (MIC) system, similar to the concept of Trifecta. The MIC was achieved when surgical margins were negative, WIT <20 min and no major complications (Clavien-Dindo≥3). The overall MIC rate was 63.1%. The MIC rate increased with surgeon's experience and decreased with complex lesion cases. Carneiro et al. [16] have investigated Trifecta rate between LPN with RAPN series. 347 patients were divided into three different groups. The patients were chronologically classified into 3 groups. The Trifecta rates increased from group 1 to group 3. (48% vs 75.6% vs 81%, *P* < 0.01). It confirmed that LPN was associated with a steep learning curve. Trifecta outcomes were significantly improved with RAPN despite increasing tumor complexity. Patients who underwent standard multiport RAPN also achieved higher Trifecta rate than those who underwent single site robot PN (42.7% vs. 25.6%, *P* = 0.021). Single site robot PN could be an alternative option for patients with decreased tumor size, low PADUA and RENAL scores, and without renal sinus or collecting system involvement [17]. Zargar, et al. [6] investigated the Trifecta rate and ‘Optimal outcomes’ of RAPN with LPN for T1a renal masses. There was a significantly higher rate of achieving Trifecta (70% vs 33%) for the RAPN group. They also reported the rate of achievement of ‘Optimal outcome’, also described as ‘Pentafecta’. It was also higher in the RAPN group (38.5% vs 24.1%). EBL, operating time, R.E.N.A.L. nephrometry score of 10–12 and tumor size were negative predictors of the accomplishment of Trifecta. Robotic technique was a positive predictor of the achievement of Trifecta. EBL, Charlson Comorbidity Index (CCI), tumor size and preoperative eGFR were predictors for accomplishment of ‘Optimal outcome’. In comparison to their findings of tumor size being one of the predictors of Pentafecta, our study did not demonstrate the difference in the rate of achieving Pentafecta between T1a andT1b renal mass. However, R.E.N.A.L. nephrometry score revealed to be a significant negative predictive factor for achievement of Pentafecta in our study. In cases performed by RAPN, rather than renal mass size, complexity revealed a significant factor for long term surgical outcome. RAPN may overcome some of the technical difficulties of laparoscopic technique, with enhanced range of freedom, improved dexterity and magnified vision. These features facilitate precise tumor resection and renorrhaphy, while minimizing WIT and as a result, the Pentafecta rate could be enhanced on RAPN series irrespective of renal mass size.

Minimally invasive PN for renal masses > 4cm, T1b, has not been well established compared to <4cm tumors. There are few studies which evaluated the feasibility, oncological and functional outcomes for T1b renal masses. Gupta et al.[18] reported the technical feasibility and renal functional and oncologic outcomes with minimum 1 year follow-up of RAPN for T1b tumors. It was feasible for renal tumors greater than 4 cm with moderate or high nephrometry scores. Although there was a modest decline in renal function of the operated unit, RAPN may facilitate the resection of more challenging tumors requiring complex renal reconstruction. Petros et al. [19] compared outcomes of RAPN for tumors >4 cm compared with RAPN for tumors ≤ 4 cm. Of 445 patients, 18.7% had tumors >4 cm with a median radiographic tumor size of 5.0 cm. Patients with tumors >4 cm had a higher proportion of hilar tumors (9.8% vs 4.7%, *P* <0.001), a higher mean R.E.N.A.L. nephrometry score (8.0 vs 6.3, *P*<0.01), long WIT (24 vs 17 min, *P*<0.001), and an increased rate of collecting system repair (72.2% vs 51.6%, *P* = 0.006) compared with patients with tumors ≤ 4 cm. Functional outcomes and complications were similar between groups. Contemporary reports on trifecta, pentafecta results of RAPN are summarized (Table 5.) [5,6,16,20–22].

**Table 5:** Comparison of contemporary reports on trifecta, pentafecta results of robot-assisted partial nephrectomy.

Series	Year	Number	Tumor size	Trifecta (%)	Pentafecta (%)
Hung et al. (5)	2013	534	T1/T2 (T1a, 85%)	45–68 (RAPN+LPN) (Chronology)	(-)
Khalifeh, et al. (20)	2013	500	T1~T3a (T1a, 83%)	58 RAPN,31 LPN	(-)
Takagi,et al. (21)	2014	163	(-)	69 OPN	(-)
Lista, et al.(22)	2015	339	T1 (T1a 86.7%)	67 RAPN	(-)
Carneiro, et al. (16)	2015	347	T1a 87%	48 LPN, 57 LPN, 81 RAPN (Chronology)	(-)
Zargar, et al. (6)	2015	1831	T1a	70 RAPN, 33 LPN	38.5 RAPN, 24.1 LPN
Current study		120	T1a/T1b	65.3 T1a, 43.3 T1b RAPN	38.3 T1a, 26.7 T1b RAPN

RAPN = robot-assisted partial nephrectomy; LPN = laparoscopic partial nephrectomy; OPN = open partial nephrectomy

Nevertheless, there has been no published data with regards to the rate of Pentafecta for renal masses > 4cm. Our study showed that there was no statistically significant difference in the Pentafecta outcome between T1a and T1b renal masses. And the R.E.N.A.L. nephrometry score (L score) was noted to be the predictor of Pentafecta achievement.

In this study, we have several limitations. First, this study was not a prospective randomized study. To eliminate the drawbacks of retrospective analysis, our study was conducted using 1:1 propensity score matching. Second, the total cohort numbers were decreased due to 1:1 matching with relatively small T1b renal mass patients. Third, the various definition of Trifecta and Pentafecta are not externally validated. Despite these limitations, this study values on adjustment of the concept ‘Pentafecta’ in larger (T1b) renal mass series treated with RAPN in clinical practice for the assessment of RAPN outcomes including long term renal function.

## Conclusions

In conclusion, Pentafecta is an effective tool in the assessment of long term perioperative, oncological, functional outcomes. Although the Pentafecta rate was lower with higher R.E.N.A.L nephrometry score, it was comparable between T1a and T1b renal masses in cases of RAPN. Therefore, RAPN is a feasible modality for patients with larger renal mass (T1b) with excellent long term outcomes. R.E.N.A.L nephrometry score, especially L score predicts the achievement of Pentafecta. Further research for validated scoring system of Pentafecta should be performed for the assessment of RAPN outcome.

## References

[1] Ljungberg B, Bensalah K, Canfield S, Dabestani S, Hofmann F, Hora M, et al EAU guidelines on renal cell carcinoma: 2014 update. Eur Urol. 2015;67(5):913–24. 10.1016/j.eururo.2015.01.005 25616710

[2] Borghesi M, Schiavina R, Gan M, Novara G, Mottrie A, Ficarra V. Expanding utilization of robotic partial nephrectomy for clinical T1b and complex T1a renal masses. World J Urol. 2013;31(3):499–504. 10.1007/s00345-013-1095-2 23645411

[3] Choi JE, You JH, Kim DK, Rha KH, Lee SH. Comparison of perioperative outcomes between robotic and laparoscopic partial nephrectomy: a systematic review and meta-analysis. Eur Urol. 2015;67(5):891–901. 10.1016/j.eururo.2014.12.028 25572825

[4] Aboumarzouk OM, Stein RJ, Eyraud R, Haber GP, Chlosta PL, Somani BK, et al Robotic versus laparoscopic partial nephrectomy: a systematic review and meta-analysis. Eur Urol. 2012;62(6):1023–33. 10.1016/j.eururo.2012.06.038 22771266

[5] Hung AJ, Cai J, Simmons MN, Gill IS. "Trifecta" in partial nephrectomy. J Urol. 2013;189(1):36–42. 10.1016/j.juro.2012.09.042 23164381

[6] Zargar H, Allaf ME, Bhayani S, Stifelman M, Rogers C, Ball MW, et al Trifecta and optimal perioperative outcomes of robotic and laparoscopic partial nephrectomy in surgical treatment of small renal masses: a multi-institutional study. BJU Int. 2015;116(3):407–14. 10.1111/bju.12933 25220543

[7] Komninos C, Shin TY, Tuliao P, Kim DK, Han WK, Chung BH, et al Robotic partial nephrectomy for completely endophytic renal tumors: complications and functional and oncologic outcomes during a 4-year median period of follow-up. Urology. 2014;84(6):1367–73. 10.1016/j.urology.2014.08.012 25440824

[8] Levin A, Stevens PE. Summary of KDIGO 2012 CKD Guideline: behind the scenes, need for guidance, and a framework for moving forward. Kidney Int. 2014;85(1):49–61. 10.1038/ki.2013.444 24284513

[9] Clavien PA, Barkun J, de Oliveira ML, Vauthey JN, Dindo D, Schulick RD, et al The Clavien-Dindo classification of surgical complications: five-year experience. Ann Surg. 2009;250(2):187–96. 10.1097/SLA.0b013e3181b13ca2 19638912

[10] Levey AS, Bosch JP, Lewis JB, Greene T, Rogers N, Roth D. A more accurate method to estimate glomerular filtration rate from serum creatinine: a new prediction equation. Modification of Diet in Renal Disease Study Group. Ann Intern Med. 1999;130(6):461–70. 10075613 10.7326/0003-4819-130-6-199903160-00002

[11] Levey AS, Coresh J, Balk E, Kausz AT, Levin A, Steffes MW, et al National Kidney Foundation practice guidelines for chronic kidney disease: evaluation, classification, and stratification. Ann Intern Med. 2003;139(2):137–47. 12859163 10.7326/0003-4819-139-2-200307150-00013

[12] Kim DK, Komninos C, Kim L, Rha KH. Robot-assisted Partial Nephrectomy for Endophytic Tumors. Curr Urol Rep. 2015;16(11):76 10.1007/s11934-015-0552-4 26373545

[13] Maddox M, Mandava S, Liu J, Boonjindasup A, Lee BR. Robotic partial nephrectomy for clinical stage T1b tumors: intermediate oncologic and functional outcomes. Clin Genitourin Cancer. 2015;13(1):94–9. 10.1016/j.clgc.2014.07.011 25176501

[14] Takagi T, Mir MC, Campbell RA, Sharma N, Remer EM, Li J, et al Predictors of precision of excision and reconstruction in partial nephrectomy. J Urol. 2014;192(1):30–5. 10.1016/j.juro.2013.12.035 24373802

[15] Porpiglia F, Bertolo R, Amparore D, Fiori C. Margins, ischaemia and complications rate after laparoscopic partial nephrectomy: impact of learning curve and tumour anatomical characteristics. BJU Int. 2013;112(8):1125–32. 10.1111/bju.12317 23937616

[16] Carneiro A, Sivaraman A, Sanchez-Salas R, Di Trapani E, Barret E, Rozet F, et al Evolution from laparoscopic to robotic nephron sparing surgery: a high-volume laparoscopic center experience on achieving 'trifecta' outcomes. World J Urol. 2015;33(12):2039–44. 10.1007/s00345-015-1552-1 25869814

[17] Komninos C, Shin TY, Tuliao P, Yoon YE, Koo KC, Chang CH, et al R-LESS partial nephrectomy trifecta outcome is inferior to multiport robotic partial nephrectomy: comparative analysis. Eur Urol. 2014;66(3):512–7. 10.1016/j.eururo.2013.10.058 24275311

[18] Gupta GN, Boris R, Chung P, Linehan WM, Pinto PA, Bratslavsky G. Robot-assisted laparoscopic partial nephrectomy for tumors greater than 4 cm and high nephrometry score: feasibility, renal functional, and oncological outcomes with minimum 1 year follow-up. Urol Oncol. 2013;31(1):51–6. 10.1016/j.urolonc.2010.10.008 21292511 PMC3123423

[19] Petros F, Sukumar S, Haber GP, Dulabon L, Bhayani S, Stifelman M, et al Multi-institutional analysis of robot-assisted partial nephrectomy for renal tumors >4 cm versus ≤ 4 cm in 445 consecutive patients. J Endourol. 2012;26(6):642–6. 10.1089/end.2011.0340 22050488

[20] Khalifeh A, Autorino R, Hillyer SP, Laydner H, Eyraud R, Panumatrassamee K, et al Comparative outcomes and assessment of trifecta in 500 robotic and laparoscopic partial nephrectomy cases: a single surgeon experience. J Urol. 2013;189(4):1236–42. 10.1016/j.juro.2012.10.021 23079376

[21] Takagi T, Mir MC, Campbell RA, Sharma N, Remer EM, Li J, et al Assessment of outcomes in partial nephrectomy incorporating detailed functional analysis. Urology. 2014;84(5):1128–33. 10.1016/j.urology.2014.07.008 25239254

[22] Lista G, Buffi NM, Lughezzani G, Lazzeri M, Abrate A, Mistretta A, et al Margin, ischemia, and complications system to report perioperative outcomes of robotic partial nephrectomy: a European Multicenter Observational Study (EMOS project). Urology. 2015;85(3):589–95. 10.1016/j.urology.2014.09.068 25733270

